# Prognostic Value of Yes-Associated Protein 1 (YAP1) in Various Cancers: A Meta-Analysis

**DOI:** 10.1371/journal.pone.0135119

**Published:** 2015-08-11

**Authors:** Zhenqiang Sun, Ruiwei Xu, Xiayu Li, Weiguo Ren, Chunlin Ou, Qisan Wang, Han Zhang, Xuemei Zhang, Jian Ma, Haijiang Wang, Guiyuan Li

**Affiliations:** 1 Key Laboratory of Carcinogenesis of Ministry of Health and Key Laboratory of Carcinogenesis and Cancer Invasion of Ministry of Education, Cancer Research Institute, Central South University, Changsha, Hunan, China; 2 Department of Gastrointestinal Surgery, Affiliated Tumor Hospital, Xinjiang Medical University, Urumqi, Xinjiang, China; 3 Department of Epidemiology and Health Statistics, School of Public Health, Central South University, Changsha, Hunan, China; 4 Department of Infection Control, Affiliated Tumor Hospital, Xinjiang Medical University, Urumqi, Xinjiang, China; 5 Hunan Key Laboratory of Nonresolving Inflammation and Cancer and Disease Genome Research Center, The Third Xiangya Hospital, Central South University, Changsha, Hunan, China; 6 Hunan Provincial Tumor Hospital and The Affiliated Tumor Hospital of Xiangya Medical School, Central South University, Changsha, Hunan, China; INRS, CANADA

## Abstract

**Background:**

Yes-associated protein 1 (YAP1) is an effector of Hippo pathway, which is critical for regulating organ size, cell proliferation and tumor growth in mammals. Many previous studies have explored the relationship between YAP1 and various types of cancer. However, these studies were limited by the small samples size and the findings were inconsistent among them. Therefore, a meta-analysis was conducted to assess the association between YAP1 and malignancies.

**Methods:**

A systematic literature search was conducted for eligible studies in the PubMed, Corchane Library, Web of Knowledge, EMBASE and CBM disc databases from inception to August 1^st^ 2014. After heterogeneity analysis, pooled harzad ratio (*HR*) with 95% confidence interval (95%*CI*) using both fixed and random effect models were estimated in STATA 10.0. Meta regression analysis, subgroup analysis and sensitivity analysis were performed to explore the potential sources of heterogeneity and to evaluate the robustness of the result. Publication bias was assessed by Egger’s test and funnel plot.

**Results:**

A total of 21 unique articles from 2009 to 2014, comprising 2983 patients, were analyzed in the meta-analysis. The association of YAP1 expression and overall survival time (OS) was evaluated in 20 studies including 2067 patients. Positive YAP1 showed poorer OS (HR = 1.826; 95% CI = 1.465–2.275; *p* <0.002). For evaluating disease-free survival time (DFS), 10 studies with 1139 patients were analyzed. Positive YAP1 indicated worse DFS (HR = 2.114; 95%CI = 1.406–3.179; *p* <0.001). Subgroup analysis showed that both positive nuclear YAP1 (HR = 1.390, 95% CI: 0.810–2.400, *p* = 0.729) and up-regulation overall YAP1 (HR = 2.237, 95% CI: 1.548–3.232, *p* <0.001) had poorer OS for patients with malignancies. Similarly, both positive nuclear YAP1 (HR = 3.733, 95% CI: 1.469–9.483, *p* = 0.001) and up-regulation overall YAP1 (HR = 1.481, 95% CI: 1.163–1.886, *p* = 0.554) showed worse DFS. The patients with urogenital system cancer had the poorest OS (HR = 2.133, 95% CI: 1.549–2.937, *p* = 0.020). The patients with alimentary system cancer had the most significant impact on DFS (HR = 1.879, 95% CI: 1.537–2.297, *p* <0.001).

**Conclusion:**

Both overall and nuclear YAP1 overexpression are intimately associated with adverse OS and DFS in numerous cancers, suggesting that YAP1 may act as a potential therapeutic targets of these malignancies in the future.

## Introduction

The Hippo pathway is an important signaling pathway controlling organ size and regulating cell proliferation and apoptosis, and dysfunction of this pathway often contributes to development and tumorigenesis [1, 2]. YAP1 is a downstream target of the Hippo pathway and plays a role as a transcription co-activator [3]. Restriction of YAP1 transcriptional activity is the principal mechanism of growth and tumor suppression by the Hippo pathway. The role of YAP1 in cancer development still remains controversial. Many previous studies have reported elevated YAP1 protein levels in various types of cancer, such as colorectal cancer (CRC), gastric cancer, human hepatocellular carcinoma (HCC) etc. YAP1 has often been described as an oncogene, which usually leads to a poor prognosis. Wang [4] found that YAP expression was closely associated with pTNM stage, nodal status, tumor status and cyclin D1 overexpression in CRC, respectively. In addition, YAP expression was also related with short overall survival (OS). Xia [5] showed that YAP expression was associated with poor ovarian cancer patient survival and high YAP expression level was positively correlated with TEAD4 gene expression.

Meanwhile, some different researchers argued that YAP1 could also be regarded as a tumor suppressor gene in some malignancies [6,7], which generally benefits cancer prognosis. Barry [8] indicated that complete loss of YAP could predicted worse patient survival and was associated with high grade, stage IV disease, compared to YAP positive groups. He said YAP could act to restrict Wnt signaling independently.

Thus, it is necessary to clarify the relationship between YAP1 and malignancies. In the present study, by using eligible relevant literatures, the first meta-analysis was conducted to achieve a precise evaluation of YAP1 prognostic value in various cancers.

## Materials and Methods

### Literature search strategy

The review protocol of this study was not preregistered. Electronic searches using PubMed, Corchane Library, Web of Knowledge with English language, EMBASE and CBM disc with Chinese language were used to identify studies on YAP1 positive expression in patients with carcinomas published from inception to August 1st, 2014. The following keywords “cancer”, “carcinoma”, “neoplasm”, “tumor, malignancy”, “hippo”, “yap1”, “yes-associated protein”, “survival” and “prognostic” (variably combined), were used in searching the databases listed above. References from all selected articles were also searched for additional eligible studies. (Search Strategy for PubMed was shown in S1 Table). For those reports on the same sample, we included the studies with more information for meta-analysis. As to studies without sufficient data, we sent emails to the corresponding authors for request,

### Study Inclusion/Exclusion Criteria

Studies included in this meta-analysis were defined as: (a) published studies with full text to measure YAP1 positive expression in the patients with any type of carcinoma by immunohistochemistry or other possible methods; (b) endpoints were OS and disease free survival (DFS) or contained survival curves; (c) studies reported a hazard ratio (HR) estimates with 95% confidence intervals (CI) or the HR with 95% CI could be estimated sufficiently; (d) the most recent or the most complete reports were included if the same author reported results from the same population; (e) searching was limited to human studies in English and Chinese.

Studies were excluded based on the following criteria: (a) review articles, laboratory articles or letters, (b) studies provided no information on survival outcomes or survival curves; (c) studies from one author and the studies brought into the repeated samples from the same patients.

### Data extraction

Studies were selected by two authors (Sun and Zhang) independently based on the inclusion criteria listed above. Any discrepancies were adjudicated by discussion to reach a consensus on all of the items. Data were collected from each publication on the first author’s name, year of publication, country of origin, age of patients, tumor type, tumor grade, TNM stage, histological differentiation, staining location, HR estimation (if both univariate and multivariate analyses were performed, HR were extracted from multivariate analyses).

### Statistical analysis

HR and 95% CI were used to combine as the effective value. If HR and 95% CI were not reported in the articles, we calculated HRs and their 95% CIs by using the data of observed deaths/cancer recurrences [9]. If only Kaplan—Meier curves were available, the data were extracted from Kaplan-Meier curves read by Engauge Digitizer version 4.1 (http://digitizer.sourceforge.net/) based on Tierney described previously [10]. The pooled HRs was examined using the Z-test. Heterogeneity among studies was measured by the Q-statistic test and I-square statistic test [11,12]. Fixed effects pooled HRs were estimated using Mantel-Haenszel method if *p* <0.05, and the DerSimonian and Laird method method was used to estimate random effects if *p* >0.05 [13,14]. Subgroup analysis was stratified by ethinicity, YAP1 staining location and systems that the carcinoma belonging to. We also carried out sensitivity analysis to evaluate the influence of a single study on the overall effect estimate by excluding one study at a time. Potential publication bias was assessed by Begg’s funnel plot and Egger’s linear regression. Meta analyses were performed using Stata 10.0 (Stata Corp, College Station, TX).

## Results

### Study characteristics and meta-analysis database

**Fig 1:**
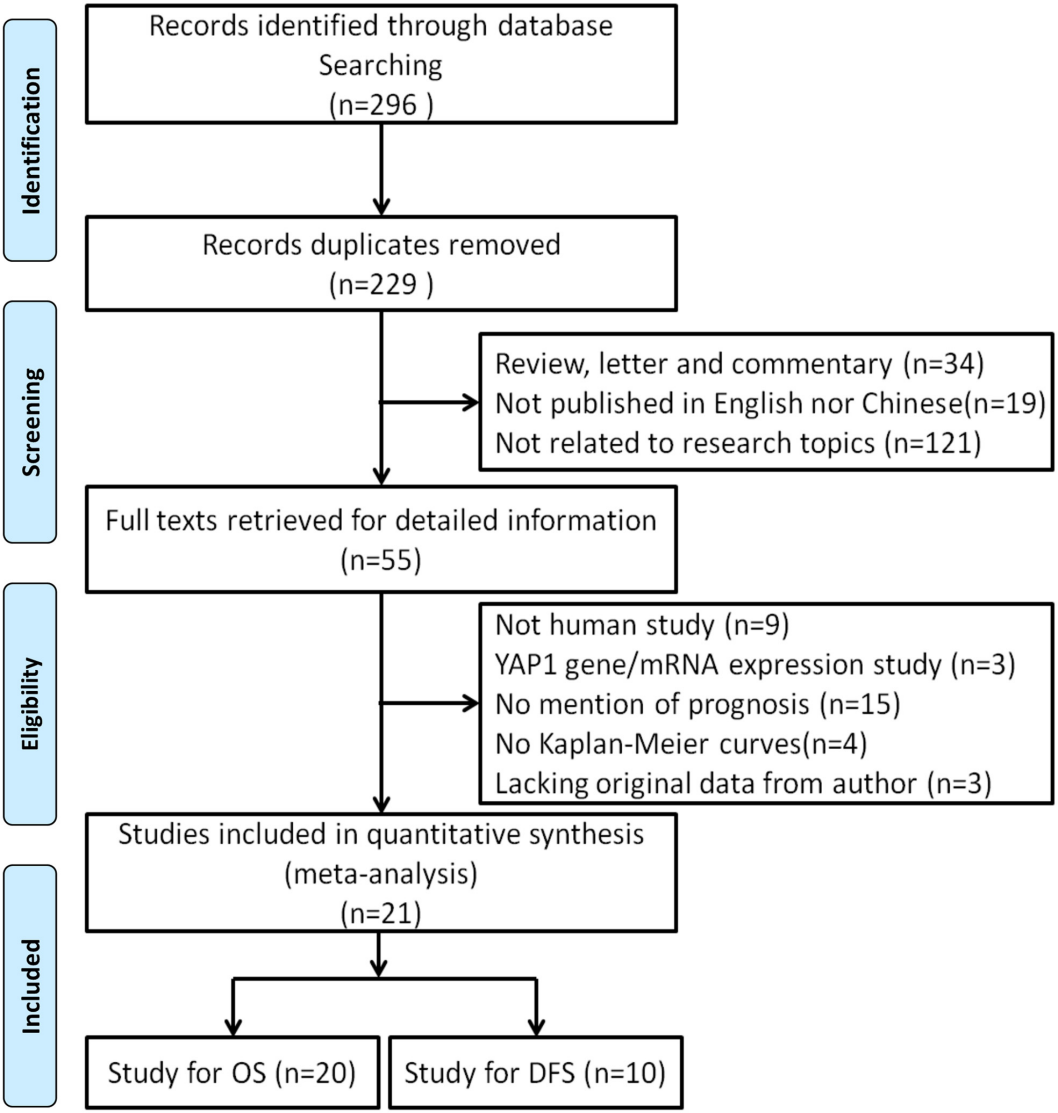
Flow chart of identification process for eligible studies

A total of 229 potentially relevant publications were retrieved after the initial database searches, and 21 observational studies met the predefined inclusion criteria comprising 2983 patients for final analysis. A flow diagram of the study selection process is presented in Fig 1. The major characteristics of the 21 eligible studies were reported in Tables 1 and 2. The studies were conducted in 5 countries (China, Japan, Korea, France and United States) and published from 2009 to 2014. Cancer types of the patients included esophageal squamous cell carcinoma, gastric carcinoma, ovarian carcinoma, cervical adenocarcinoma carcinoma, colon carcinoma, hepatocellular carcinoma, bladder carcinoma, lung carcinoma, breast cancer and endometrial carcinoma. Overall, 20 studies were performed on the association between YAP1 positive expression and OS [4,5,8,15–31], and 10 studies on DFS [17–19,21,24,25,29–36]. For OS, YAP1 positive expression was detected by nuclear staining in 10 studies [5,8,15,18,19,21,23,24,26,31], by nuclear and cytoplasmic staining (overall YAP1 expression) in 11 studies [4,16,17,20,22,25,27–30,32]. For DFS, YAP1 positive expression was detected by nuclear staining in 7 studies [17–19,21,24,31,32], by nuclear and cytoplasmic staining in 3 studies [25,29,30].

**Table 1:** Characteristics of studies included in the meta-analysis for OS.

Author	Year	Country	Age	Follow-up time(months)	No.(Negative/Positive)	Tumor type	TNM stage(Negative/Positive)	T stage(Negative/Positive)	N stage(Negative/Positive)	M stage(Negative/Positive)	Tumor grade(Negative/Positive)	HR estimation	Staining location	HR (95% CI)
Xu [30]	2009	China	mean±SD: 53.9±12.2	125*	110/67	Hepatocellular Carcinoma	I/II/III: 29/42,20/34,18/34	-	-	M0: 67/110	G1/G2/G3: 20/13,32/57,9/21	Reported in text	YAP expression	2.148(1.255~3.677)
Muramatsu [23]	2010	Japan	-	Median: 19 range (1~103)	51/69	Esophageal squamous cell carcinoma	-	-	-	-	-	Reported in text	YAP nuclear expression	1.764(1.081~2.882)
Song [24]	2012	Korea	<65 years/≥65 years:15/68	About 78*	162/61	Gastric carcinoma	II/III: 68/25,94/36	T1/T2/T3/T4: 4/1,22/9,64/19/72/32	N0/N1/N2/N3: 29/14,43/9,37/21,53/17	M0: 162/61	G1/G2/G3/others: 7/5,46/16,63/30,46/10	Reported in text	YAP nuclear expression	1.233(0.789~1.927)
Yeo [31]	2012	Korea	(Negative/Positive)Mean±SD:68.88±8.88/69.61±9.61	More than 60*	104/38	Esophageal squamous cell carcinoma	I/II/III-IV: 21/2,34/15,49/21	-	-	-	G1+G2/G3: 91/27,13/11	Data extrapolated	YAP nuclear expression	2.006(1.052~3.825)
Wang [28]	2013	China	≤60 years/>60 years: 46/46	More than 60*	31/61	Lung carcinoma	I/II-IV: 20/20,11/41	T1/T2-4: 7/23,24/38	N0/N1-2: 24/30,7/31	-	-	Data extrapolated	YAP expression	1.52(0.900~2.500)
Luo [22]	2013	China	Mean±SD: 66.1±3.7,Range:60~73	60*	16/35	Gastric carcinoma	I-II/III-IV: 14/19,3/20	T1-T2a/T2b-T4: 14/22,3/17	N0/N1-3: 15/21,2/18	-	G1+G2/G3: 10/30,6/10	Data extrapolated	YAP expression	1.82(0.440~7.690)
Xu [29]	2013	China	≤50 years/ >50 years: 35/45	42*	47/33	Hepatocellular Carcinoma	I/II/III/IV: 11/3,24/15/,12/14,0/1	-	-	-	G1/G2/G3: 14/6,29/23,4/4	Data extrapolated	YAP expression	6.67(2.860~16.670)
Wang [4]	2013	China	<60 years /≥60 years: 57/82	More than 60*	66/73	Colorectal carcinoma	I/II/III/IV: 21/7,28/30,14/25,3/11	T1-2/T3-4: 24/15,42/58	N0/N1-3: 43/37,17/36	M0/M1: 63/62,3/11	G1/G2/G3: 5/8,38/45,23/20	Reported in text	YAP expression	1.910(1.110~3.290)
Wang [27]	2013	China	≤40 years/ 40~60 years /≥60 years: 19/66/83	60*	46/122	Colon carcinoma	I/II/III/IV: 4/8,23/37,15/68,4/9	T1/T2/T3/T4: 1/0,5/9,29/105,2/8	N0/N1/N2: 31/43,13/66,2/13	M0/M1: 40/112,6/10	G1/G2/G3: 56/20,52/15,14/11	Reported in text	YAP expression	1.62(1.150 ~2.270)
Liu [20]	2013	China	(Negative/Positive)Median:≤62 years:54/57,>62 years:46/56	Mean:86.36,range(56~120)	100/113	Bladder carcinoma	-	T1/T2-4: 19/23,29/53	N0/N+: 96/99,4/14		G1/G2/G3: 49/28,29/40,22/45	Reported in text	YAP expression	5.500 (2.460~12.300)
Barry [8]	2013	America	-	120*	195/56	Colorectal carcinoma	-	-	-	-	-	Data extrapolated	YAP nuclear expression	1.39(0.840~2.290)
Liu [21]	2013	China	(Negative/Positive)<45years:18/4,≥45 years:13/7	About 100*	31/11	Cervical adenocarcinoma Carcinoma	-	-	N0/N+: 23/9,8/2	-	G1/G2/G3: 7/3,14/6,10/2	Data extrapolated	YAP nuclear expression	1.02(0.170~8.330)
Kim [17]	2013	Korea	(Negative/Positive)≤60 years:41/20,>60years:54/29	125	84/48	Colorectal carcinoma	I-II/III-IV: 53/22,52/27	-	-	-	G1/G2/G3: 27/17,56/29,12/3	Reported in text	YAP nuclear expression	1.206(0.606~2.400)
Kim [18]	2013	Korea	<50 years/≥50 years:43/109	125	85/67	Hepatocellular carcinoma	-	-	-	-	G1+G2+G3: 124/28	Data extrapolated	YAP nuclear expression	1.39(0.810~2.40)
Xia [5]	2014	China	-	80*	total YAP: 30/15, nuclear YAP:35/10	Ovarian carcinoma	-	-	-	-	-	Data extrapolated	YAP nuclear expression, YAP expression	1.59(0.580~1.670); 4.34(4.380~11.100)
Tsujiura [26]	2014	America	(Negative/Positive) Median:62.8(40~89)/64.2 (44~82)	About 66*	54/66	Endometrial carcinoma	I-II/III-IV: 53/56,1/10	-	-	M0/M1: 53/57,1/9	-	Data extrapolated	YAP nuclear expression	0.12(0.010~3.830)
Kim [19]	2014	Korea	(Negative/Positive)≤50 years:377/259, >50 years:26/16	140*	636/42	Breast carcinoma	I-II/III: 423/30,213/12	T1/T2-3: 305/18,331/24	N0/N1: 377/23,259/19	M0: 636/42	G1+G2/G3: 449/4,223/2	Reported in text	YAP nuclear expression	2.073(0.973~4.416)
Touil [25]	2014	France	-	About 110*	39/10	Colon carcinoma	-	-	-	-	-	Data extrapolated	YAP expression	3.85(1.470~10.000)
Jeong [16]	2014	America	Median 59,Range:22~80	60*	-	Ovarian Cancer	-	-	-	-	-	Reported in text	YAP expression	1.66(1.100~2.530)
Han [15]	2014	China	Mean±SD:51.77±1.93	25*	.12/27	Hepatocellular carcinoma	I-II/III+IV: 5/10,7/17	-	-	-	-	Reported in text	YAP expression	0.054(0.007~0.413)

**Table 2:** Characteristics of studies included in the meta-analysis for DFS. Note: No.: Number of patients for survial analysis; T: Depth of invasion; N: lymph node metastasis; M: distant metastasis, G:Histological differentiation, well differentiated (G1), moderately differentiated (G2), poorly differentiated (G3), undifferentiated (G4);

Author	Year	Country	Age	Follow-up time(months)	No.(Negative/Positive)	Tumor type	TNM stage(Negative/Positive)	T stage(Negative/Positive)	N stage(Negative/Positive)	M stage(Negative/Positive)	Tumor grade(Negative/Positive)	HR estimation	Staining location	HR (95% CI)
Xu [30]	2009	China	mean±SD: 53.9±12.2	125*	110/67	Hepatocellular Carcinoma	I/II/III: 29/42,20/34,18/34	-	-	M0: 67/110	G1/G2/G3: 20/13,32/57,9/21	Reported in text	YAP expression	1.653(1.081~2.528)
Hall [32]	2010	America	Median: 59.8	60*	41/24	Ovarian carcinoma	-	-	-	-	-	Data extrapolated	YAP nuclear expression	3.3(0.750~14.260)
Song [24]	2012	Korea	<65 years/≥65 years:15/68	About 78*	162/61	Gastric carcinoma	II/III: 68/25,94/36	T1/T2/T3/T4: 4/1,22/9,64/19/72/32	N0/N1/N2/N3: 29/14,43/9,37/21,53/17	M0: 162/61	G1/G2/G3/others: 7/5,46/16,63/30,46/10	Reported in text	YAP nuclear expression	0.86(0.340~2.200)
Yeo [31]	2012	Korea	(Negative/Positive)Mean±SD:68.88±8.88/69.61±9.61	More than 60*	104/38	Esophageal squamous cell carcinoma	I/II/III-IV: 21/2,34/15,49/21	-	-	-	G1+G2/G3: 91/27,13/11	Data extrapolated	YAP nuclear expression	1.69(1.190~2.440)
Xu [29]	2013	China	≤50 years/ >50 years: 35/45	42*	47/33	Hepatocellular Carcinoma	I/II/III/IV: 11/3,24/15/,12/14,0/1	-	-	-	G1/G2/G3: 14/6,29/23,4/4	Data extrapolated	YAP expression	5.88(3.125~11.110)
Liu [21]	2013	China	(Negative/Positive)<45years:18/4,≥45 years:13/7	About 100*	31/11	Cervical adenocarcinoma Carcinoma	-	-	N0/N+: 23/9,8/2	-	G1/G2/G3: 7/3,14/6,10/2	Data extrapolated	YAP nuclear expression	1.72(0.170~16.670)
Kim [17]	2013	Korea	(Negative/Positive)≤60 years:41/20,>60years:54/29	125	84/48	Colorectal carcinoma	I-II/III-IV: 53/22,52/27	-	-	-	G1/G2/G3: 27/17,56/29,12/3	Reported in text	YAP nuclear expression	1.99(0.610~6.520)
Kim [18]	2013	Korea	<50 years/≥50 years:43/109	125	85/67	Hepatocellular carcinoma	-	-	-	-	G1+G2+G3: 124/28	Data extrapolated	YAP nuclear expression	1.867(0.841~4.147)
Kim [19]	2014	Korea	(Negative/Positive)≤50 years:377/259, >50 years:26/16	140*	636/42	Breast carcinoma	I-II/III: 423/30,213/12	T1/T2-3: 305/18,331/24	N0/N1: 377/23,259/19	M0: 636/42	G1+G2/G3: 449/4,223/2	Reported in text	YAP nuclear expression	2.073(0.973~4.416)
Touil [25]	2014	France	-	About 110*	39/10	Colon carcinoma	-	-	-	-	-	Data extrapolated	YAP expression	5.88(2.940~12.500)

*: read from survial curves

### Methodological Quality of the Studies

Study quality were assessed by two authors (Sun and Xu) independently using the Newcastle-Ottawa quality assessment scale (NOS). The scores of the included studies ranged from 6 to 7 (with a mean of 6.19). S2 Table summarizes the quality scores of each item of studies.

### Quantitative synthesis

#### Overall analysis

**Fig 2:**
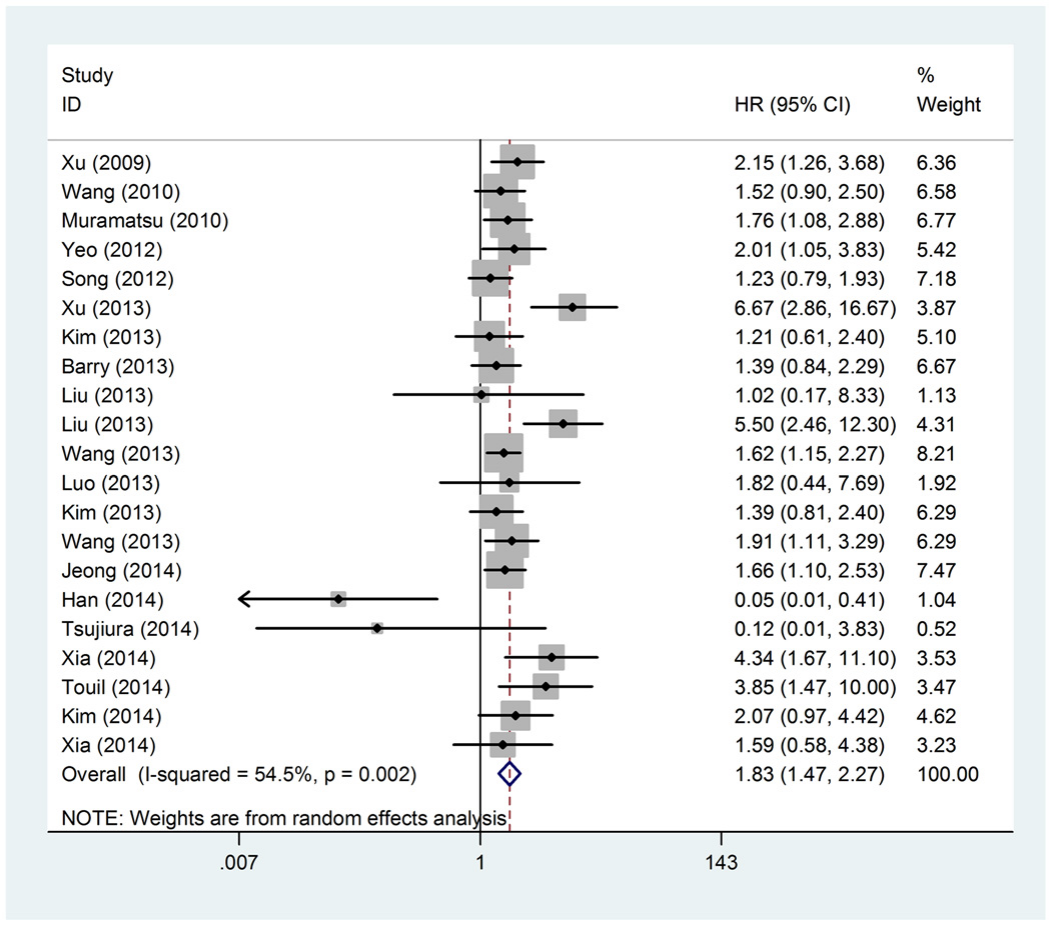
Forest plot of the hazard ratio (HR) for the association of YAP1 expression with overall survival. (Random-effects model). The HRs of individual studies are shown as squares, with the size proportional to the weight of each study in the overall estimate; 95% CIs are shown as horizontal lines. The pooled HRs and their 95% CIs are shown as a dashed vertical line and a diamond, respectively.

**Fig 3:**
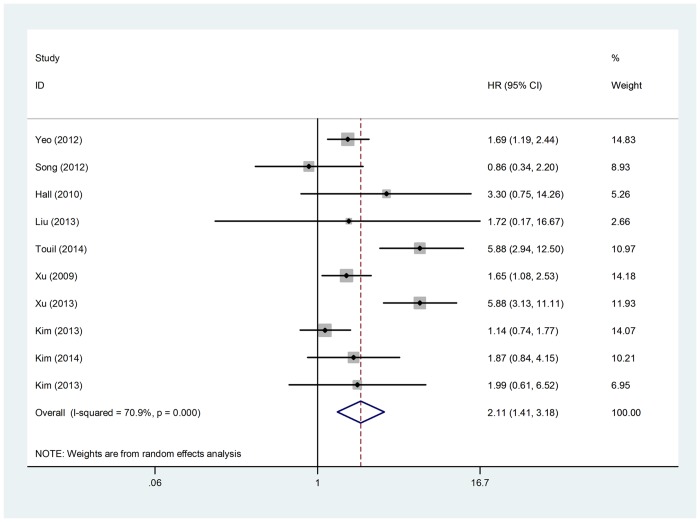
Forest plot of the hazard ratio (HR) for the association of YAP1 expression with disease-free survival. (Random-effects model). The HRs of individual studies are shown as squares, with the size proportional to the weight of each study in the overall estimate; 95% CIs are shown as horizontal lines. The pooled HRs and their 95% CIs are shown as a dashed vertical line and a diamond, respectively.

In total, there were 20 studies including 2067 patients to evaluate the relation of YAP1 expression and OS [4,5,8,15–31]. Heterogeneity was found between studies (Q = 44.00, I^2^ = 54.50% and *p* = 0.002). Hence, a random effect model was applied to calculate a pooled HR and its 95% CI. Meta analysis showed that positive YAP1 expression was associated with poor OS (HR = 1.826; 95% CI = 1.465–2.275; *p* <0.001). For studies evaluating DFS, 10 studies with 1139 patients were included. Pooled HR being 2.114 (95%CI 1.406–3.179, *p* <0.001) was obtained from the random effect model (Q = 30.97, I^2^ = 70.90% and *p* <0.001), suggesting that positive YAP1 expression significantly predicted worse DFS. From the above analysis, YAP1 positive expression proved to be a significant prognostic biomarker for OS and DFS (*p* <0.001), (Table 3, Figs 2 and 3).

**Table 3:** Results of overall and subgroup analyses for effects of YAP1 expression on overall and disease-free survival in cancer. Note: *P* ^a^ for Z test, *P* ^b^ for *x* ^2^-based Q test, N: number of studies include, Heterogeneity test: Q, df, Pb, I2 and 95%CI for I2.

	Categories	N	Effect model	HR	95% CI	P^a^	Q	df	P^b^	I2	95%CI for I2
Overall survival (OS)		20	Random	1.826	1.465–2.275	<0.001	44	19	0.002	0.545	0.289–0.738
Subgroup1	YAP1 total expression	11	Random	2.237	1.548–3.232	<0.001	33.13	10	<0.001	0.698	0.438–0.838
	YAP1 nuclear expression	10	Fixed	1.474	1.203–1.807	<0.001	6.11	9	0.729	0	0–0.623
Subgroup2	Asian	16	Random	1.773	1.525–2.061	<0.001	37.39	15	0.002	0.572	0.306–0.768
	Caucasian	4	Random	1.647	1.217–2.228	0.001	6.43	3	0.092	0.534	0–0.846
Subgroup3	Alimentary System	13	Random	1.673	1.427–1.961	<0.001	28.39	12	0.005	0.577	0.217–0.771
	Urogenital system	5	Random	2.133	1.549–2.937	<0.001	13.35	4	0.02	0.625	0.235–0.882
	Other System	2	Fixed	1.675	1.097–2.558	0.017	0.44	1	0.505	0	-
Subgroup4	lower score	16	Random	1.734	1.493–2.014	<0.001	33.71	15	0.006	0.525	0.22–0.746
	higher score	4	Random	1.804	1.324–2.458	<0.001	10.24	3	0.017	0.707	0.179–0.899
Disease-free survival (DFS)		10	Random	2.114	1.406–3.179	<0.001	30.97	9	<0.001	0.709	0.445–0.848
Subgroup1	YAP1 expression	3	Random	3.733	1.469–9.483	0.006	15.16	2	0.001	0.868	0.622–0.954
	YAP1 nuclear expression	7	Fixed	1.481	1.163–1.886	0.001	4.92	6	0.554	0	0–0.708
Subgroup2	Asian	8	Random	1.718	1.405–2.101	<0.001	20.11	7	0.005	0.652	0.259–0.836
	Caucasian	2	Fixed	5.255	2.745–10.061	<0.001	0.48	1	0.49	0	-
Subgroup3	Alimentary System	7	Random	1.879	1.537–2.297	<0.001	30.41	6	<0.001	0.853	0.587–0.902
	Urogenital system	2	Fixed	2.728	0.79–9.418	0.112	0.22	1	0.639	0	-
	Other System	1	-	1.867	0.841–4.146	0.125	0	-	-	-	
Subgroup4	lower score	8	Random	1.764	1.407–2.211	<0.001	20.78	7	0.004	0.663	0.286–0.841
	higher score	2	Random	2.288	1.586–23.301	<0.001	8.79	1	0.003	0.886	0

#### Meta regression analysis

Given that significant heterogeneity existed in our overall analysis, meta-regression was conducted to explore the potential factors responsible for heterogeneity. For OS analysis, the results revealed that publication years (*p* = 0.870, I2 = 55.36%), ethnicity of the objects (p = 0.834,I2 = 55.24%), HR estimation method(*p* = 0.578, I2 = 54.94%), staining location of YAP-1 (*p* = 0.139, I2 = 50.17%) and the study quality (*p* = 0.724, I2 = 55.32%) could only account minor heterogeneity in our study with the consideration of both Q-statistic test and I-square statistic test. For DFS analysis, publication years (*p* = 0.483, I2 = 71,63%), HR estimation method (*p* = 0.253, I2 = 71.75%) and the study quality (*p* = 0.463, I2 = 72.28%) could explained morderate heterogeneity, while ethnicity of the objects (*p* = 0.099, I2 = 60.79%) and staining location of YAP-1 (*p* = 0.060, I2 = 59,40%) may be the main factors contributed to the heterogeneity.

### Subgroup analysis

Subgroup analyses was performed by all four possible factors (staining location, ethnicity of the objects, tumor systems and the study quality) after the stratification of studies into four subgroups with the consideration of clinical charateristics. For studies evaluating OS, subgroup analysis by staining location demonstrated that YAP1 positive expression was significantly related with poor OS for both nuclear staining (HR: 1.474, 95% CI: 1.203–1.807, *p* = 0.729 without heterogeneity) and nuclear combining with cytoplasmic staining (HR: 2.237, 95% CI: 1.548–3.232, *p* <0.001) in patients with cancers. When grouped according to ethinicity, the pooled HR of Asians and Caucasions were 1.773 (95% CI: 1.525–2.061, *p* = 0.002 with less heterogeneity) and 1.647 (95% CI: 1.217–2.228, *p* = 0.092), respectively. When stratifying by different systems that the carcinoma belonging to, heterogeneity still existed, patients with carcinomas belonging to urogenital system had the poorest OS (HR = 2.133, 95% CI: 1.549–2.937, *p* = 0.020), compared with alimentary system (HR = 1.673, 95% CI: 1.427–1.961, *p* = 0.005) and others (lung and breast cancer) (HR = 1.675, 95% CI: 1.097–2.558, *p* = 0.505). In study quality subgroup, the intimate relation between YAP1 positive expression and poor OS was observed in both low NOS score studies (HR: 1.734, 95% CI: 1.493–2.014, *p* = 0.006) and high NOS score studies (HR: 1.804, 95% CI: 1.324–2.458, *p* = 0.017), having significant heterogeneity (Table 3 and S1 Fig).

Of the studies reporting the prognostic value of positive YAP1 expression for DFS, when stratifying by staining location, both positive overall (nuclear and cytoplasmic) YAP1 expression (HR: 3.733, 95% CI: 1.469–9.483, *p* = 0.001) and positive nuclear YAP1 expression (HR: 1.481, 95% CI: 1.163–1.886, *p* = 0.554 without heterogeneity) indicated worse cancer DFS. Being grouped according to ethinicity, Asian patients (HR = 1.718, 95% CI: 1.405–2.101, *p* = 0.005) and non-Asian patients (HR = 5.255, 95% CI: 2.745–10.061, *p* = 0.490 without heterogeneity) both showed statistically significant results. When grouped according to different carcinoma systems, patients with carcinoma of alimentary system had significant impacts on DFS (HR = 1.879, 95% CI: 1.537–2.297, *p* <0.001), but not for urogenital system carcinoma patients (HR = 2.728, 95% CI: 0.790–9.418, *p* = 0.112 without heterogeneity). Subgroup analysis by study quality suggested that the close relationship between YAP1 positive expression and poor OS was revealed in both low NOS score studies (HR: 1.764, 95% CI: 1.407–2.211, *p* <0.001) and high NOS score studies (HR: 2.288, 95% CI: 1.586–23.301, *p* = 0.003). (Table 3 and S2 Fig).

### Sensitivity analyses

**Fig 4:**
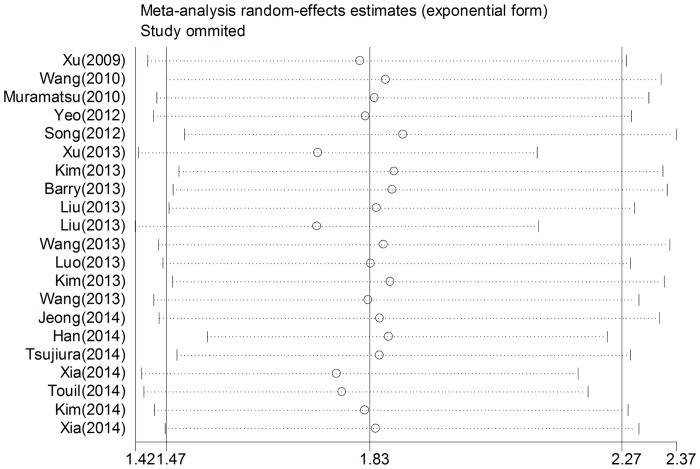
Sensitivity analysis based on stepwise omitting one study at a time for overall survival.

**Fig 5:**
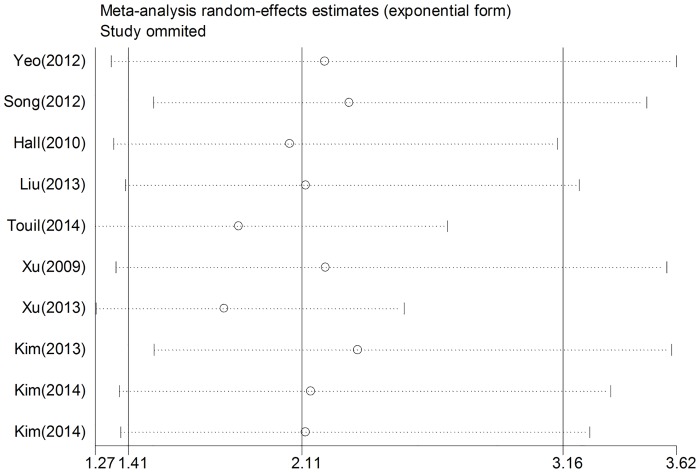
Sensitivity analysis based on stepwise omitting one study at a time for Disease-free survival

Analysis of sensitivity was conducted to evaluate the robustness of association between YAP1 positive expression and survival outcome (OS and DFS). Statistical heterogeneity and the pooled HR were analyzed by excluding one study at each time. Results revealed that no individual study significantly changed the pooled HRs of our meta-analysis for both OS and DFS, indicating that the results were stable (Figs 4 and 5).

### Publication bias

**Fig 6:**
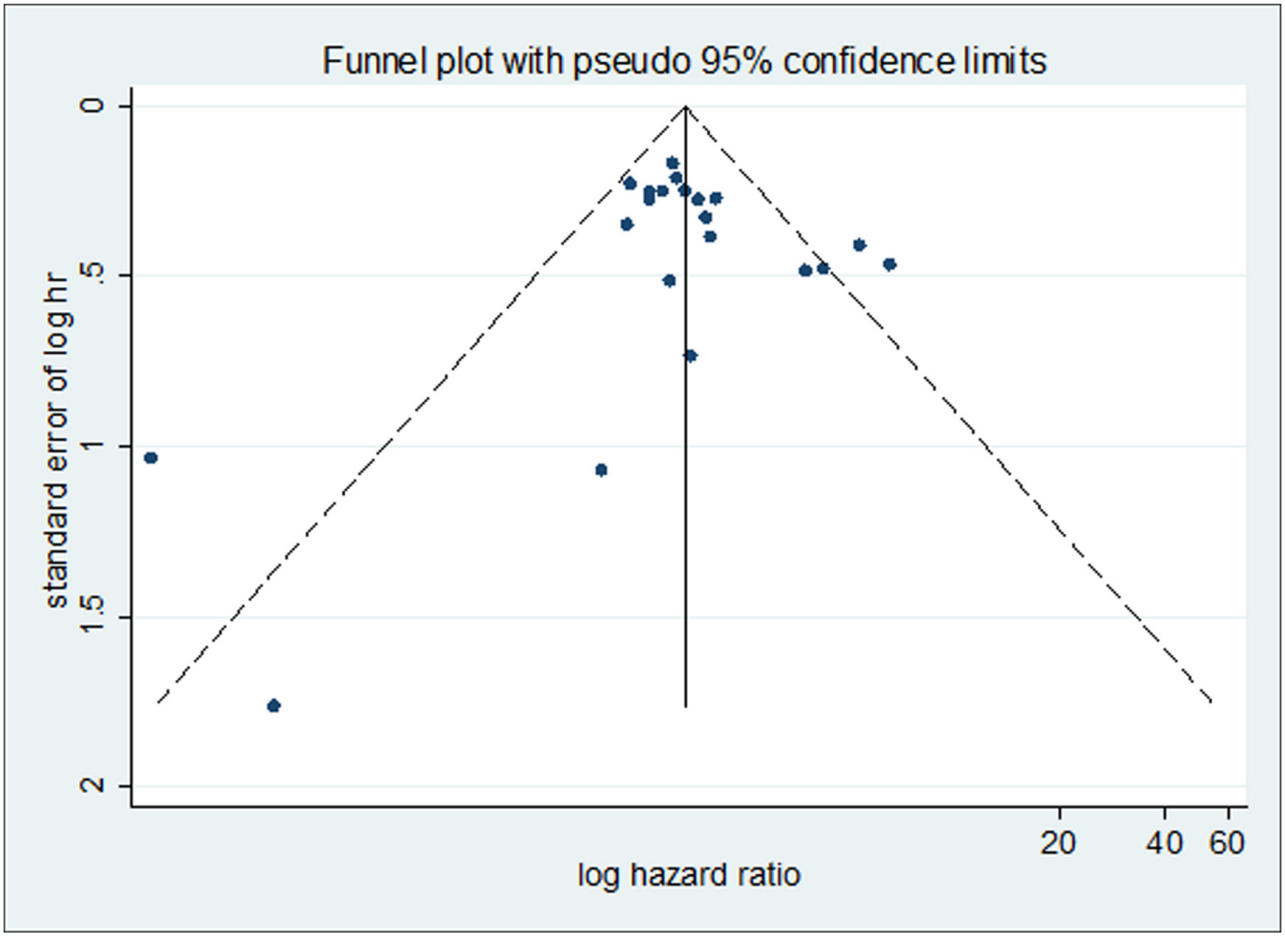
Begg’s funnel plot for the evaluation of potential publication bias on overall estimate of overall survival. The vertical line in the funnel plot indicates the random-effects summary estimate, while the sloping lines indicate the expected 95% confidence intervals for a given standard error, assuming no heterogeneity between studies. Each study is represented by a circle.

**Fig 7:**
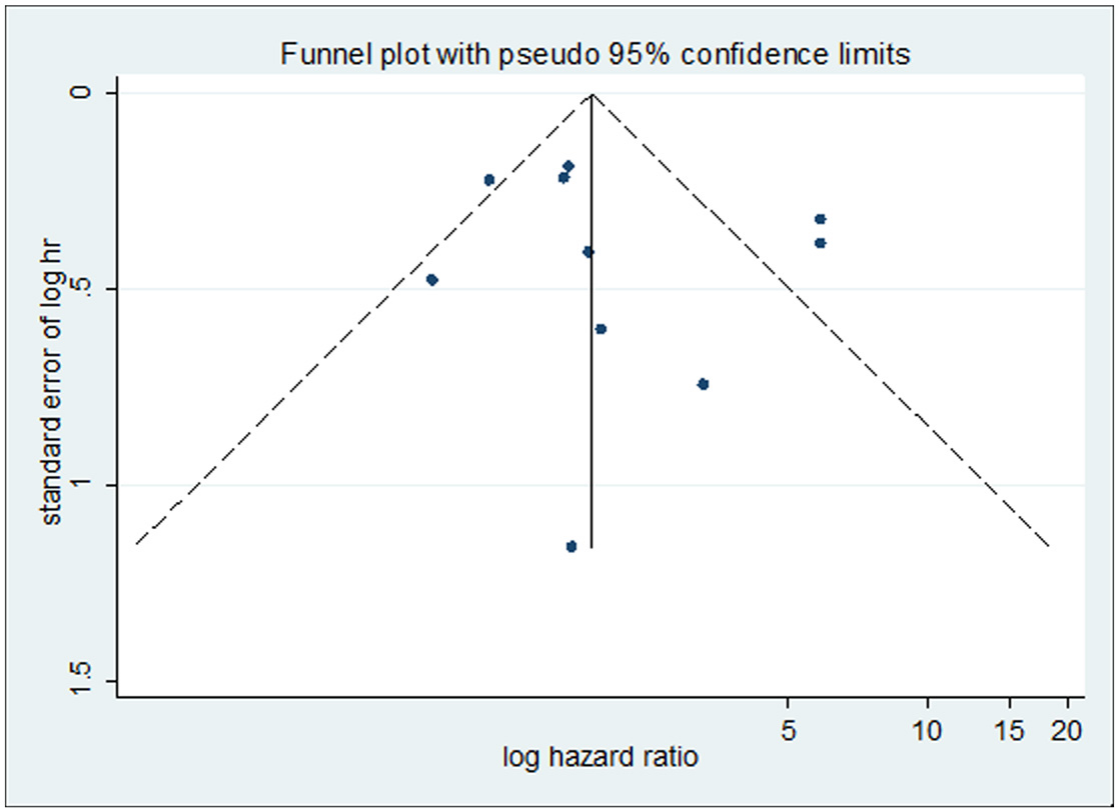
Begg’s funnel plot for the evaluation of potential publication bias on overall estimate of Disease-free survival. The vertical line in the funnel plot indicates the random-effects summary estimate, while the sloping lines indicate the expected 95% confidence intervals for a given standard error, assuming no heterogeneity between studies. Each study is represented by a circle.

Begg’s funnel plots and Egger’s test were conducted to exam publication bias of the studies on the summary of OS and DFS. The shape of the funnel plots was symmetrical which indicated that there was no publication bias. Additionally, the results of the Egger’s test (*P* = 0.958 for OS; *p* = 0.455 for DFS) provided statistical evidence of funnel plot symmetry, also suggesting that no publication bias was found for the positive YAP1 expression on OS and DFS (Figs 6 and 7).

## Discussion

Hippo signaling is an evolutionarily conserved pathway that controls organ size by regulating cell proliferation, apoptosis, epithelial-mesenchymal transition, stem cell self renewal and cross talk with other pathways, such as TGF-β/SMAD [33], epidermal growth factor receptor signaling [34, 35], Hedgehog pathway [36], PI3K/mTOR [37], Wnt/β-catenin [38] and Notch pathway [39]. YAP1, locating in the 11q22, is a critical component of the size-controlling Hippo signaling pathway. YAP1 is a well-characterized downstream transcriptional co-activator of Hippo pathway that interacts with various transcription factors and modulates their transcriptional activities in cell nuclear. When Hippo pathway is activated in mammals, YAP1 is phosphorylated by large tumor suppressor 1/2 at the Serine127 site through inhibiting their localization into nucleus [40–42]. This process leads to inactivation of YAP1.

YAP1 can influence multiple signaling pathways to promote Hippo pathway to play roles. In recent years, plenty of studies have been carried out to explore how YAP1 effects, especially on tumorigenesis, tumor development and cancer prognosis. YAP1 has been reported to have several oncogenic properties in some malignancies, including anchorage independent growth, epithelial-mesenchymal transition, and resistance to apoptosis [43,44]. The YAP1 characters above benefit the growth of tumor cells and are bad for prognosis in many malignancies. Meanwhile, YAP1 has ever been defined as a tumor suppressor that induces apoptosis in response to DNA damage in collaboration with p73 and promyelocytic leukemia [6,45,46] in a few cancers. In some studies on the same type of cancers through different mechanism, different angle conclusions were acquired.

YAP1 was found elevated in many cancers, such as gastric cancer [17,19,47], ovarian cancer [5,16,32], esophageal squamous cell carcinoma [23,31], cervical carcinoma [21], urothelial carcinoma of the bladder [20], non-small-cell lung cancer [28] et al. The prognostic indicator role of YAP1 expression in patients with various cancers has been analyzed in the previous studies. The present study, a meta-analysis including 2067 patients for OS and 1139 patients for DFS from 21 studies, explored the prognostic role of YAP1 expression in patients with malignancies. There was between-study heterogeneity in both OS studies (I2 = 54.5%) and DFS studies (I2 = 70.9%). After meta regression analysis and subgroup analysis, studies with different systems the carcinoma belonged to exhibited obvious heterogeneity in OS analysis group. Meanwhile, heterogeneity from studies of inequal quality could be tested in both OS and DFS group. The possible reason for this heterogeneity may due to the diversity of patients’ characteristics in the baseline and the quality of studies that different researches carried out. Although we have recognized some of the heterogeneity in our study, considerable heterogeneity remained present, indicating that not all sources of heterogeneity could be accounted for. By considering this, we applied random effect model to minimize the effect. Our result supported that the positive YAP1 expression could indicate both poor OS and poor DFS in patients with carcinomas. In the subgroups according to staining location, different ethinicities, cancer systems and the study quality, our results indicated that the positive YAP1 expression was statistically significantly associated with the poor prognostic outcomes. Some researchers said that up-regulation YAP could improve cellular proliferation [48]. Xenograft mice transplanted with a YAP-overexpressing breast cancer cell line enhanced tumor formation and growth [49]. In recent years, more mechanisms have been found in deeper researches. Nuclear YAP1 can bind ErbB4, TEAD and RUNX2 [50–56] inducing cell proliferation, oncogeneic transformation, and the epithelial-tomesenchymal transition (EMT) et al, which leading to poor cancer prognosis.

Some limitations of our meta-analysis should be considered in interpreting the results. Firstly, lacking the original data of the reviewed studies limited the power of our study. Using Engauge Digitizer to extract survival data from Kaplan-Meier curves contained some potential bias, which seemed to be less reliable than obtaining HR directly from published statistics. Secondly, our results were based on unadjusted estimates, while a more precise analysis should be conducted if more detailed individual data were available, which would allow for an adjusted estimate by other factors such as age and other factors. Lacking the information for the data analysis may cause serious confounding bias. Finally, though a total of 21 studies were included, the total sample number of 2067 patients for OS and 1139 patients for DFS might be not enough. These limited samples might result in a relative high risk of bias in this meta-analysis. In spite of these limitations, our meta-analysis also had some advantages. To begin with, the substantial numbers of cases and controls were pooled from different studies, which significantly increased the statistical power of the analysis. What’s more, the quality of studies included in current meta-analysis was satisfactory and met our inclusion criterion. In addition, so far our study was the first meta-analysis that attempted to assess the prognostic role of YAP1 expression in patients with various malignancies.

In summary, our meta-analysis suggests that the positive YAP1 expression can statistically contribute to poor OS and DFS in patients with carcinoma. However, larger studies using standardized unbiased methods, enrolling quantitative YAP1 expression measurements, with more detailed individual data are needed.

## Supporting Information

S1 Fig Subgroup-analysis on the relation between YAP1 expression and overall survival (OS). (ZIP)

S2 Fig Subgroup-analysis on the relation between YAP1 expression and disease free survival (DFS). (ZIP)

S1 Table Search Strategy for PubMed. (DOCX)

S2 Table Newcastle-Ottawa quality assessment scale (cohort studies). (DOCX)
