# Supplementary figures and images for: Prognostic Value of Yes-Associated Protein 1 (YAP1) in Various Cancers: A Meta-Analysis
Source: PLoS One. 2015 Aug 11;10(8):e0135119. doi: 10.1371/journal.pone.0135119 (PMC4532485; doi:10.1371/journal.pone.0135119)

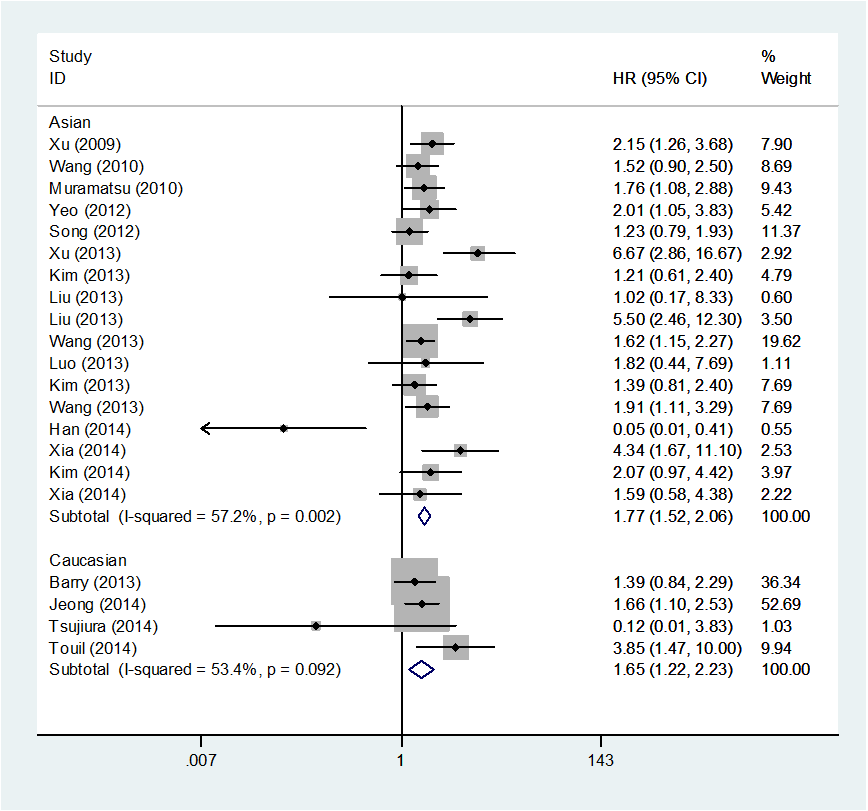

Supplement: S1 Fig — (ZIP) [file pone.0135119.s001.zip › Figure S1A.tif]

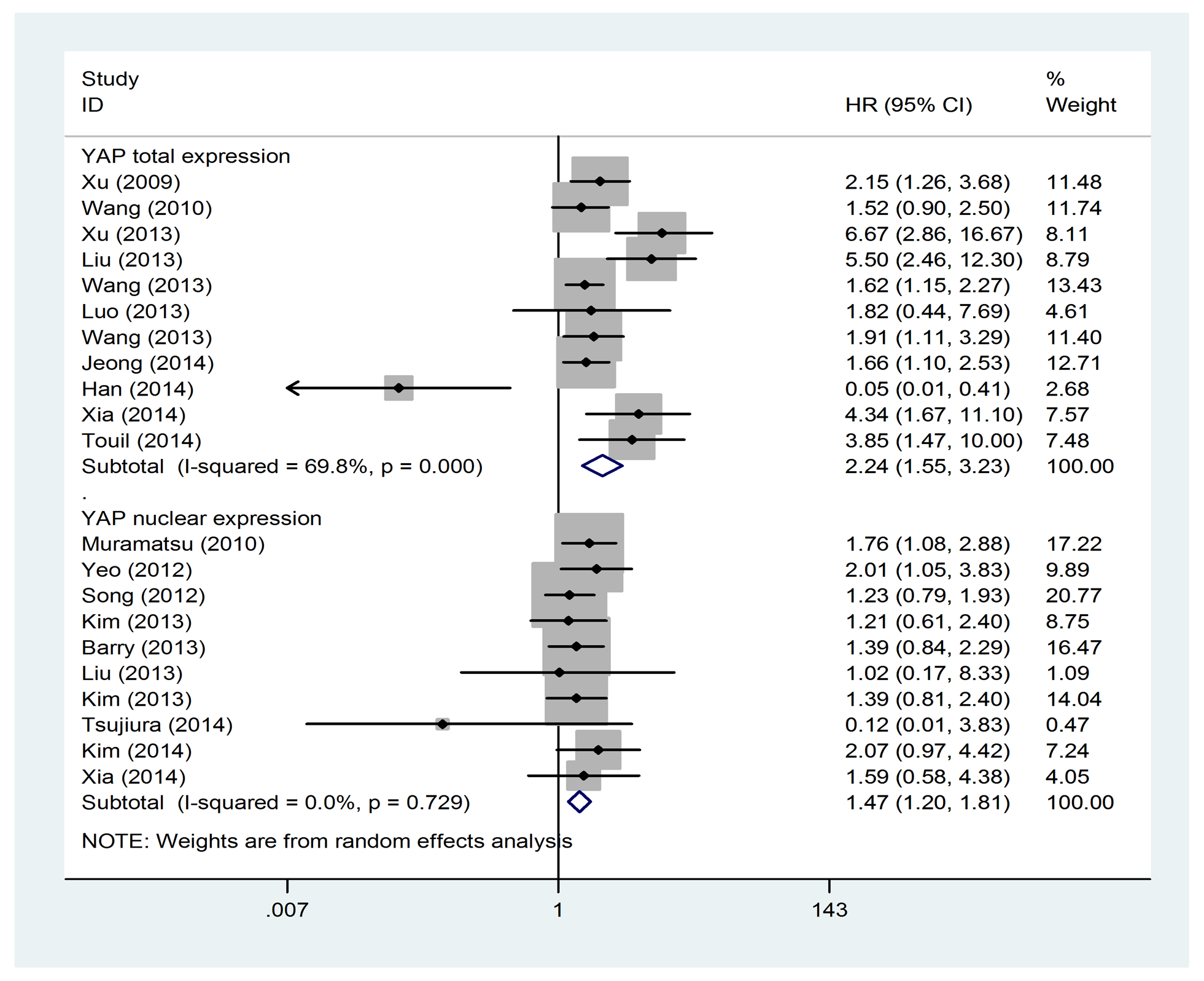

Supplement: S1 Fig — (ZIP) [file pone.0135119.s001.zip › Figure S1B.tif]

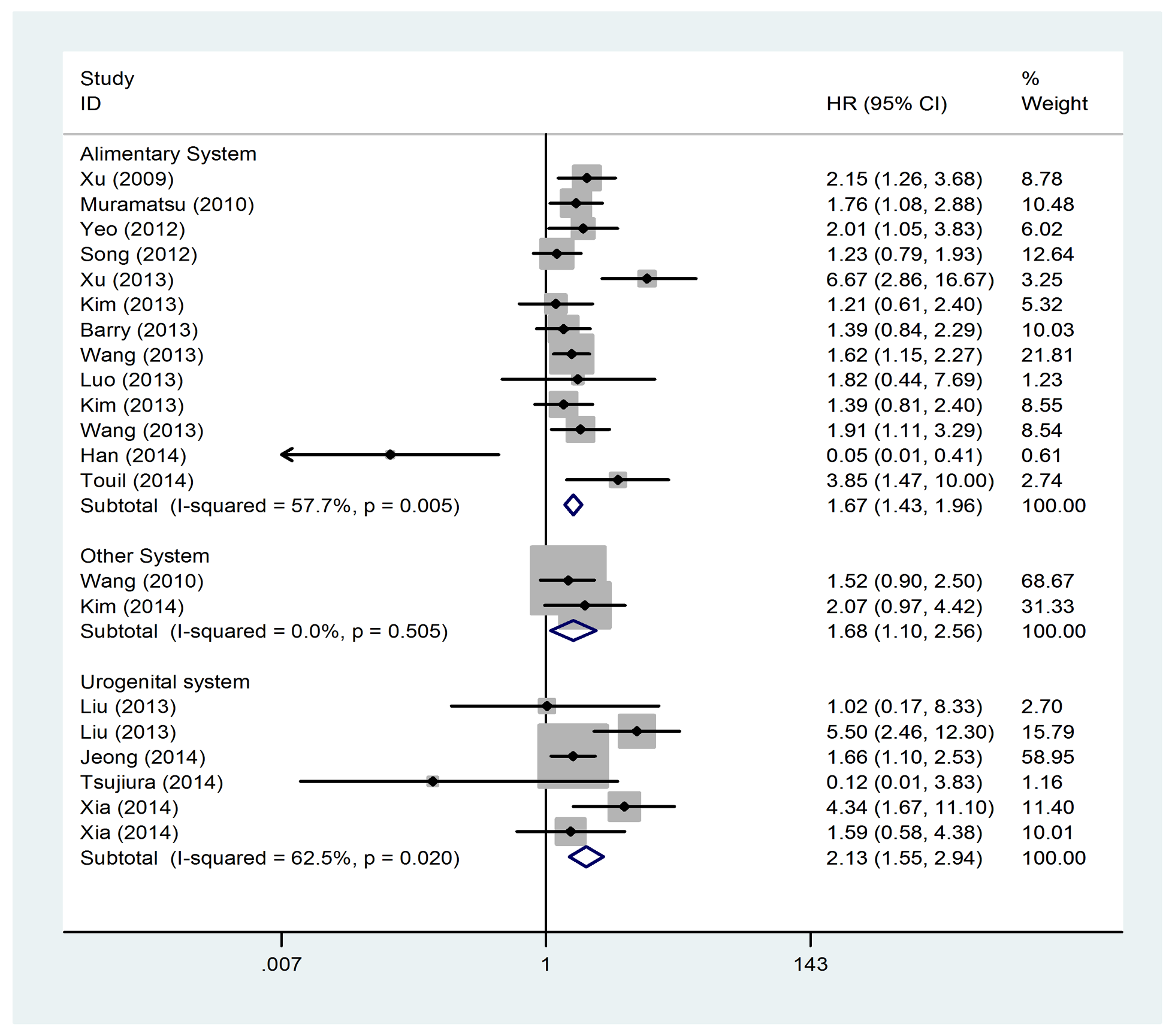

Supplement: S1 Fig — (ZIP) [file pone.0135119.s001.zip › Figure S1C.tif]

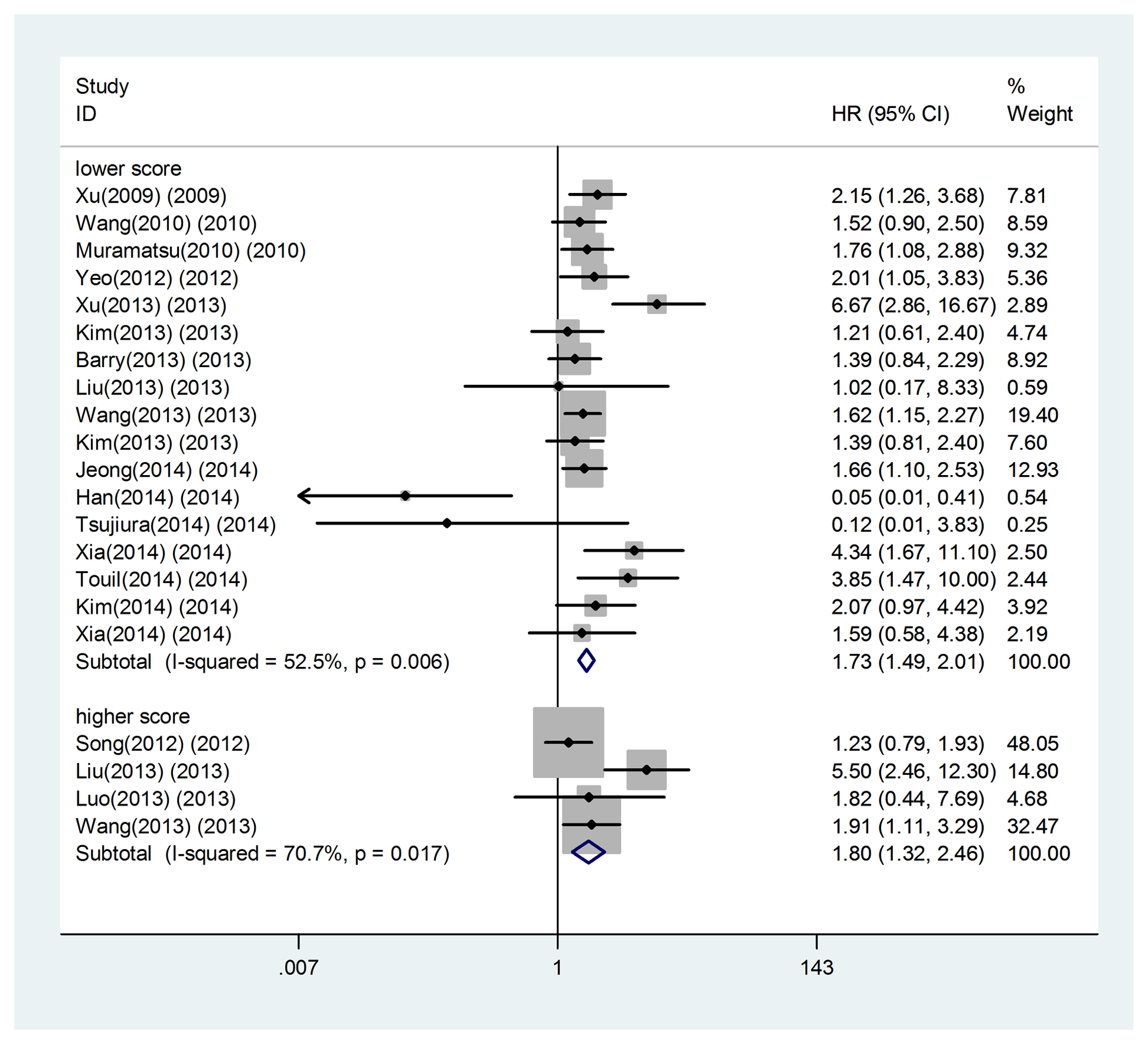

Supplement: S1 Fig — (ZIP) [file pone.0135119.s001.zip › Figure S1D.tif]

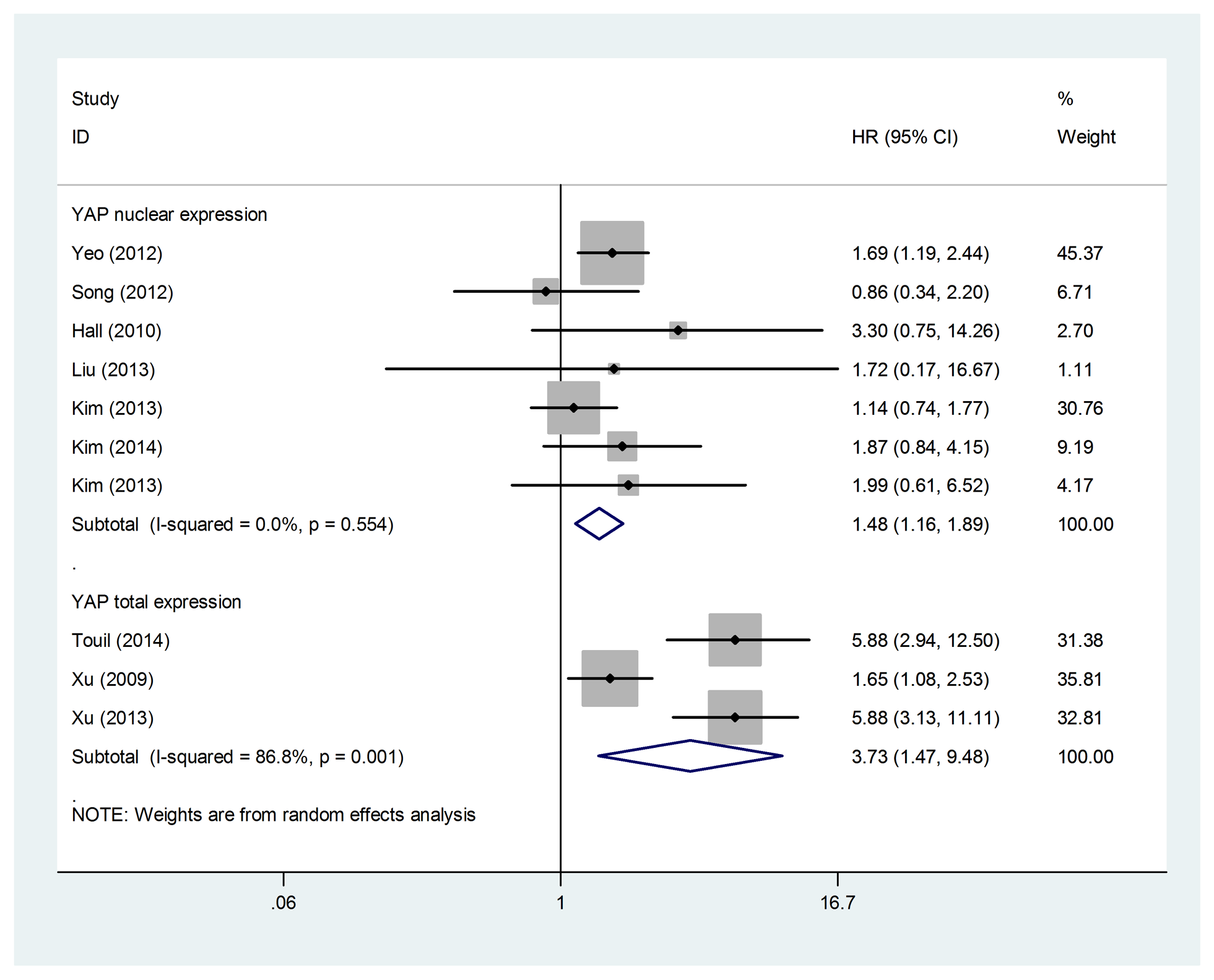

Supplement: S2 Fig — (ZIP) [file pone.0135119.s002.zip › Figure S2A.tif]

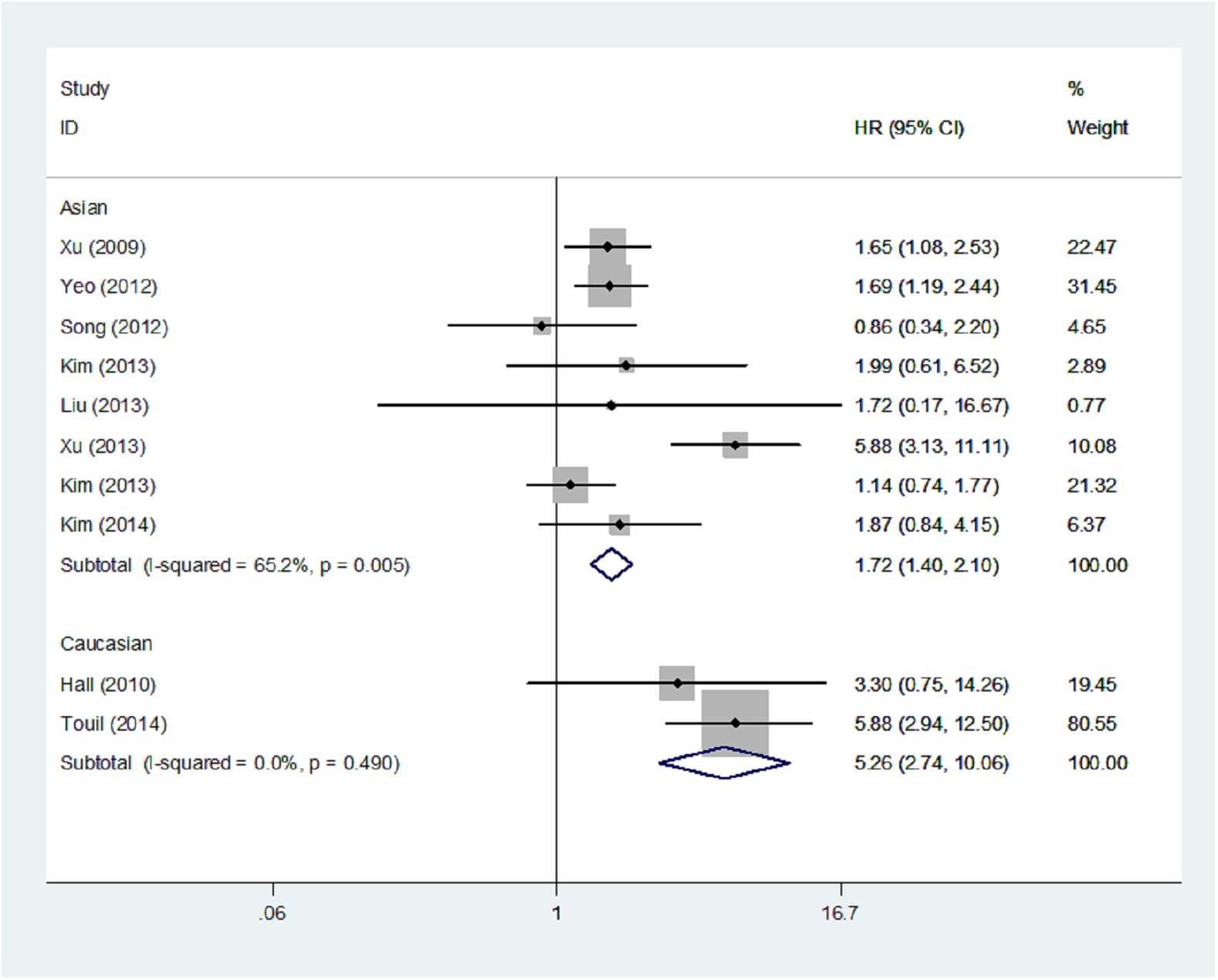

Supplement: S2 Fig — (ZIP) [file pone.0135119.s002.zip › Figure S2B.tif]

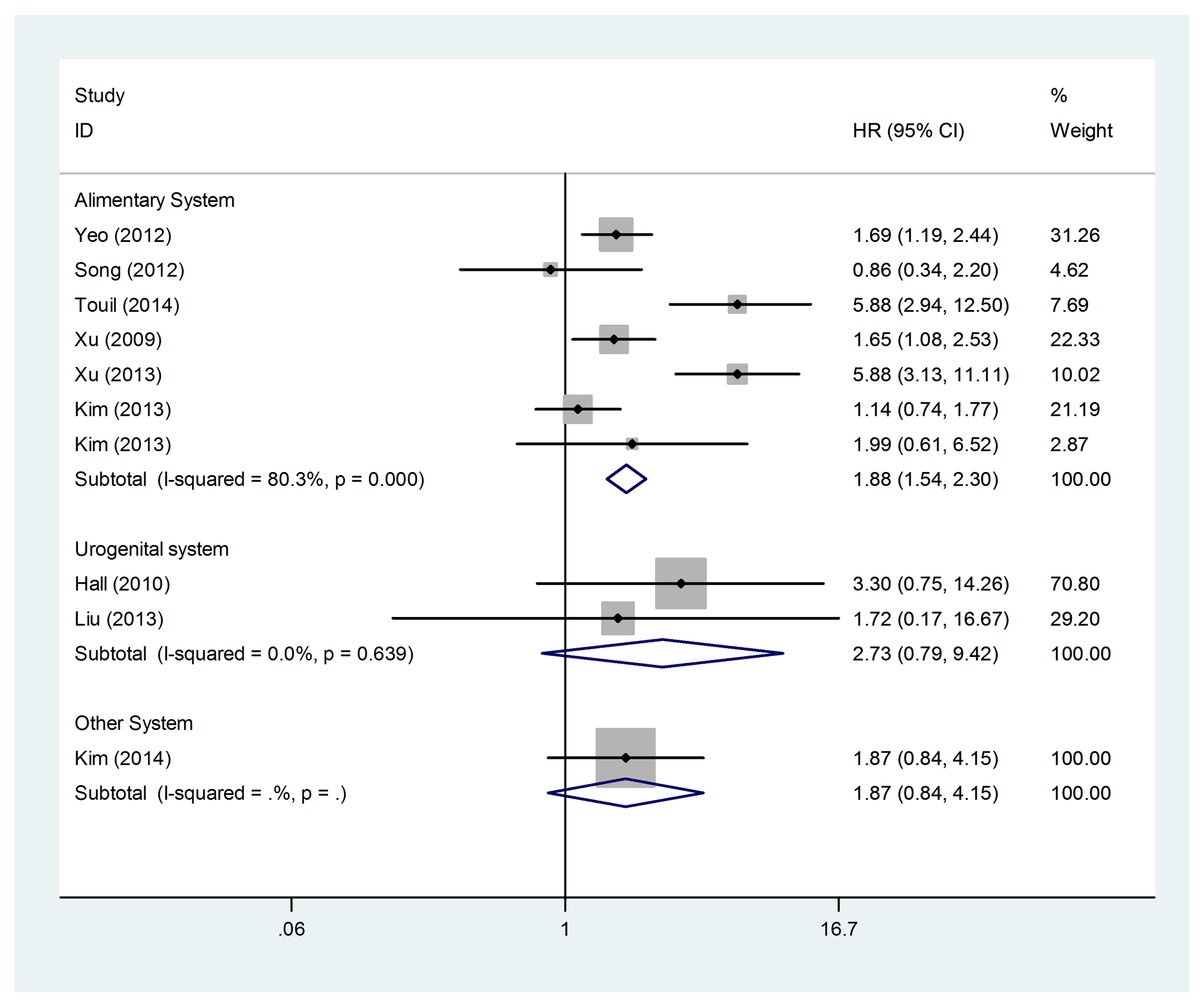

Supplement: S2 Fig — (ZIP) [file pone.0135119.s002.zip › Figure S2C.tif]

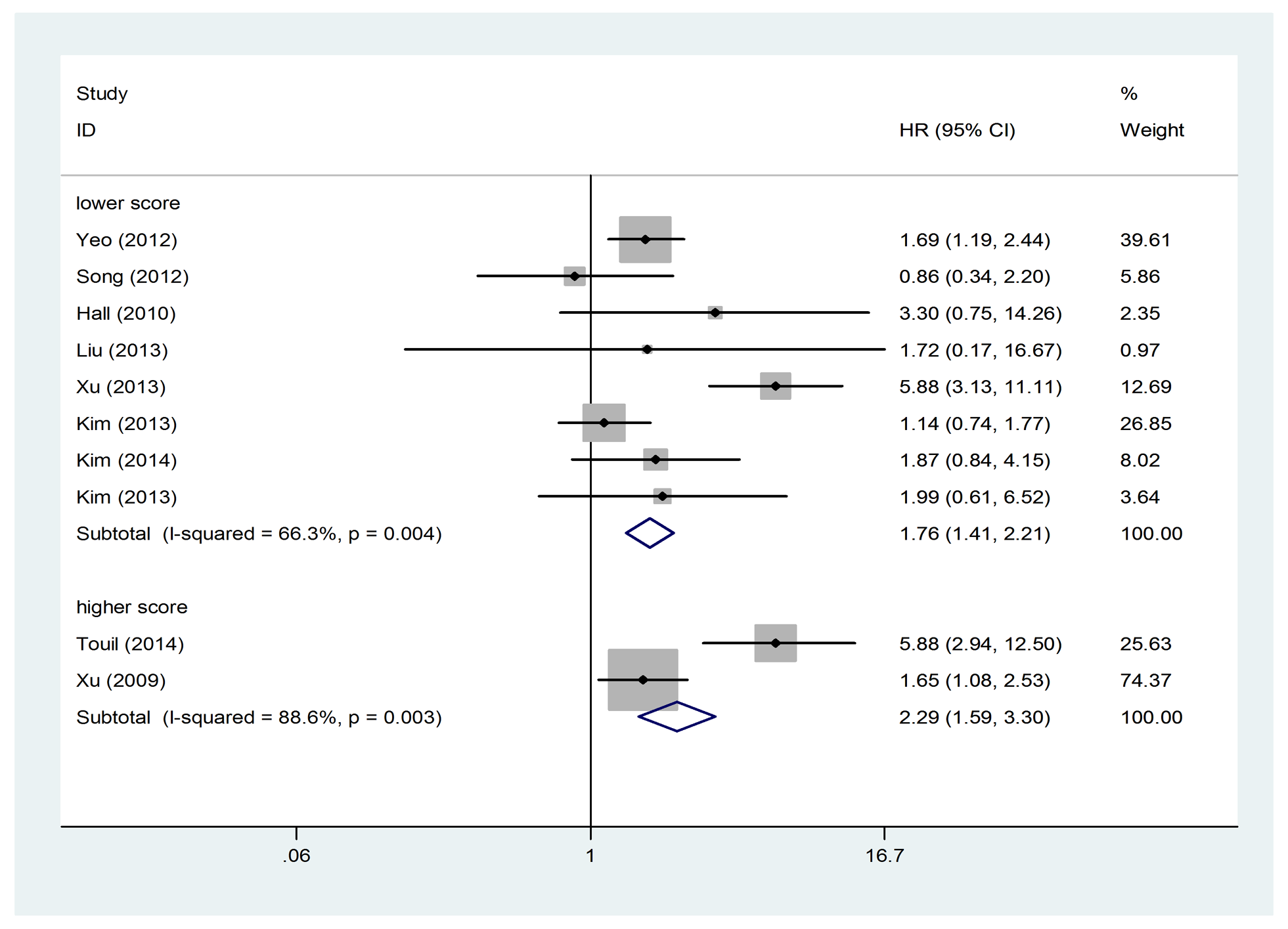

Supplement: S2 Fig — (ZIP) [file pone.0135119.s002.zip › Figure S2D.tif]
