# Supplementary material for: Prognostic Value of Yes-Associated Protein 1 (YAP1) in Various Cancers: A Meta-Analysis
Source: PLoS One. 2015 Aug 11;10(8):e0135119. doi: 10.1371/journal.pone.0135119 (PMC4532485; doi:10.1371/journal.pone.0135119)
Supplement: S1 Table — (DOCX) [file pone.0135119.s003.docx]

**S1 Table. Search Strategy for PubMed**

| # 1 " carcinoma "[MeSH Terms] OR " tumor "[MeSH Terms] OR " neoplasm"[MeSH Terms] OR "cancer "[All Fields] |
| --- |
| #2 "hippo"[MeSH Terms] |
| #3 " yes-associated protein "[MeSH Terms] OR "YAP1"[All Fields] |
| #4 " prognostic "[MeSH Terms] OR " survival "[All Fields] |
| #5 #1 AND #2 AND #3 AND #4 |
| #6 ("0001/01/01"[PDAT] : "2014/08/01"[PDAT]) |
| #7 #5AND #6 |
