# Supplementary material for: Prognostic Value of Yes-Associated Protein 1 (YAP1) in Various Cancers: A Meta-Analysis
Source: PLoS One. 2015 Aug 11;10(8):e0135119. doi: 10.1371/journal.pone.0135119 (PMC4532485; doi:10.1371/journal.pone.0135119)
Supplement: S2 Table — (DOCX) [file pone.0135119.s004.docx]

**S2 Table. Newcastle – Ottawa quality assessment scale （cohort studies）**

|  | **Selection** | | | | **Comparability** | **Outcome** | | | **Score** |
| --- | --- | --- | --- | --- | --- | --- | --- | --- | --- |
|  | **Representativeness of the  exposed cohort** | **Selection of the non-exposed cohort** | **Ascertainm-ent of exposure** | **Demonstration that outcome of interest was not present at start of study** | **Comparability of cohorts on the basis of the design or analysis** | **Assessment of outcome** | **Was follow-up long enough for outcomes to occur** | **Adequacy of follow up of cohorts** |  |
|  | **a.** truly representative of the average (describe) in the community ☆  **b.** somewhat representative of the average in the community☆  **c.** selected group of users eg nurses,volunteers  **d.** no description of the derivation of the cohort | **a.** community controls ☆  **b.** drawn from a different source  **c．**no description of the derivation of the non exposed cohort | **a.** secure record (eg surgical records)☆  **b.** structured interview  **c.** written self report  **d.** no description | **a.**  yes (endpoint) ☆  **b.** no | **a.** study controls for (select the most important factor) ☆  **b.** study controls for any additional factor (This criteria could be modified to indicate specific control for a second important factor.)☆ | **a.**independent blind assessment ☆  **b.** record linkage☆  **c.** self report  **d.** no description | **a.** yes (select an adequate follow up period for outcome of interest)☆  **b.** no | **a.** complete follow up - all subjects accounted for ☆  **b.** subjects lost to follow up unlikely to introduce bias-small number lost -> 80 % (select an adequate %) follow up, or description provided of those lost) ☆  **c.** follow up rate <_80_% (select an adequate %) and no description of those lost  **d．**no statement |  |
| **[25]** | a☆ | ☆ | ☆ |  | ☆ | b☆ | ☆ | b☆ | 7 |
| **[18]** | b☆ | ☆ | ☆ |  | ☆ | b☆ | ☆ |  | 6 |
| **[19]** | b☆ | ☆ | ☆ |  | ☆ | b☆ | ☆ |  | 6 |
| **[26]** | b☆ | ☆ | ☆ |  | ☆ | b☆ | ☆ |  | 6 |
| **[23]** | b☆ | ☆ | ☆ |  | ☆ | b☆ | ☆ |  | 6 |
| **[17]** | b☆ | ☆ | ☆ |  | ☆ | b☆ | ☆ |  | 6 |
| **[24]** | b☆ | ☆ | ☆ |  | ☆ | b☆ | ☆ | b☆ | 6 |
| **[4]** | b☆ | ☆ | ☆ |  | ☆ | a☆ | ☆ |  | 6 |
| **[22]** | b☆ | ☆ | ☆ |  | ☆ | a☆ | ☆ | b☆ | 7 |
| **[15]** | b☆ | ☆ | ☆ |  | ☆ | b☆ | ☆ |  | 6 |
| **[8]** | a☆ | ☆ | ☆ |  | ☆ | b☆ | ☆ |  | 7 |
| **[16]** | b☆ | ☆ | ☆ |  | ☆ | b☆ | ☆ |  | 6 |
| **[12]** | b☆ | ☆ | ☆ |  | ☆ | b☆ | ☆ |  | 6 |
| **[13]** | b☆ | ☆ | ☆ |  | ☆ | b☆ | ☆ |  |  |
| **[5]** | b☆ | ☆ | ☆ |  | ☆ | b☆ | ☆ |  | 6 |
| **[21]** | b☆ | ☆ | ☆ |  | ☆ | b☆ | ☆ |  | 6 |
| **[14]** | b☆ | ☆ | ☆ |  | ☆ | b☆ | ☆ |  | 6 |
| **[20]** | b☆ | ☆ | ☆ |  | ☆ | b☆ | ☆ | b☆ | 7 |
| **[11]** | a☆ | ☆ | ☆ |  | ☆ | a☆ | ☆ |  | 6 |
| **[10]** | b☆ | ☆ | ☆ |  | ☆ | b☆ | ☆ |  | 6 |
| **[27]** | b☆ | ☆ | ☆ |  | ☆ | b☆ | ☆ |  | 6 |

Note: A study can be awarded a maximum of one star for each numbered item within the Selection and Outcome categories. A maximum of two stars can be given for Comparability
